# Variability in integration of mechanisms associated with high tolerance to progressive reductions in central blood volume: the compensatory reserve

**DOI:** 10.14814/phy2.12705

**Published:** 2016-02-16

**Authors:** Robert Carter, Carmen Hinojosa‐Laborde, Victor A. Convertino

**Affiliations:** ^1^ US Army Institute of Surgical ResearchFort Sam HoustonTexas

**Keywords:** Presyncope, hypovolemia, hemorrhage, compensatory reserve

## Abstract

High tolerance to progressive reductions in central blood volume has been associated with higher heart rate (HR), peripheral vascular resistance (PVR), sympathetic nerve activity (SNA), and vagally mediated cardiac baroreflex sensitivity (BRS). Using a database of 116 subjects classified as high tolerance to presyncopal‐limited lower body negative pressure (LBNP), we tested the hypothesis that subjects with greater cardiac baroreflex withdrawal (i.e., BRS > 1.0) would demonstrate greater LBNP tolerance associated with higher HR, PVR, and SNA. Subjects underwent LBNP to presyncope. Mean and diastolic arterial pressure (MAP; DAP) was measured by finger photoplethysmography and BRS (down sequence) was autocalculated (WinCPRS) as ∆R‐R Interval/∆DAP. Down_BRS_ **(**ms/mmHg) was used to dichotomize subjects into two groups (Group 1 = Down_BRS_ > 1.0, *N* = 49, and Group 2 = Down_BRS_ < 1.0, *N* = 67) at the time of presyncope. Muscle SNA was measured directly from the peroneal nerve via microneurography (*N* = 19) in subjects from Groups 1 (*n* = 9) and 2 (*n* = 10). Group 1 (Down_BRS_ > 1.0) had lower HR (107 ± 19 vs. 131 ± 20 bpm), higher stroke volume (45 ± 15 vs. 36 ± 15 mL), less SNA (45 ± 13 vs. 53 ± 7 bursts/min), and less increase in PVR (4.1 ± 1.3 vs. 4.5 ± 2.6) compared to Group 2 (Down_BRS_ < 1.0). Both groups had similar tolerance times (1849 ± 260 vs. 1839 ± 253 sec), MAP (78 ± 11 vs. 79 ± 12 mmHg), compensatory reserve index (CRI) (0.10 ± 0.03 vs. 0.09 ± 0.01), and cardiac output (4.5 ± 1.2 vs. 4.7 ± 1.1 L/min) at presyncope. Contrary to our hypothesis, higher HR, PVR, SNA, and BRS were not associated with greater tolerance to reduced central blood volume. These data are the first to demonstrate the variability and uniqueness of individual human physiological strategies designed to compensate for progressive reductions in central blood volume. The sum total of these integrated strategies is accurately reflected by the measurement of the compensatory reserve.

## Introduction

In military and civilian trauma, hemorrhage is the leading cause of death (Bellamy 1984; Bellamy et al. 1986; Sauaia et al. 1995). Severe bleeding is accompanied by a complexly integrated response of mechanisms that control cardiac output and peripheral vascular resistance (PVR). An experimental model of progressive hemorrhage known as lower body negative pressure (LBNP) in humans (Johnson et al. 2014) that is designed to induce hemodynamic decompensation (i.e., severe hypotension and presyncope) has demonstrated that individuals with a high tolerance (HT) to central hypovolemia display higher heart rate and peripheral vasoconstriction, elevated release of vasoactive hormones, greater sympathetic nerve activity, and protection of systemic blood flow (i.e., cardiac output) (Sather et al. 1986; Convertino and Sather 2000; Convertino et al. 2012; Rickards et al. 2011). However, the relative contributions of each of these mechanisms in a homogeneous population with high tolerance to central hypovolemia have not been investigated.

The key challenge in assessing the clinical status of trauma patients with progressive hemorrhage is reflected by attempts to oversimplify measurement(s) of the condition. For instance, greater tolerance to reductions in central blood volume is associated with increased arterial–cardiac baroreflex sensitivity (BRS) withdrawal and sympathoexcitation (Cooke and Convertino 2005; Convertino et al. 2012). As such, metrics of BRS and/or sympathetic nerve activity (SNA; e.g., heart rate variability) have been used to assess pathological states, including myocardial infarction, hypertension, and congestive heart failure (Sleight 2007). Although the relationship between autonomic functions and physiological adaptation has significant variation, lower BRS or high SNA have been generally associated with negative physiological and health outcomes (Pitzalis et al. 2003; Laude et al. 2004; Murrell et al. 2007; Gouveia et al. 2008).

The inconsistencies in the ability of isolated physiological responses to predict tolerance to reduced central blood volume may reflect the complexity of integration of various compensatory mechanisms. In an attempt to evaluate such complexity, we developed an algorithm to compute the compensatory reserve index (CRI) that accurately tracks the reserve to compensate for central blood volume loss (Convertino et al. 2013, 2016; Moulton et al. 2013).

In this study, we had the opportunity to use a database of human volunteers who had been classified as HT (Convertino et al. 2013) to progressive central hypovolemia based on exposure to lower body negative pressure (LBNP) to presyncope. Because vagal withdrawal is associated with tolerance to central hypovolemia (Cooke and Convertino 2005; Convertino et al. 2012), we used BRS at the time of presyncope to identify two subgroups of HT subjects. We sought to better understand the relationship between independent and integrated compensatory mechanisms involved in dictating tolerance to reduced central blood volume in a subpopulation of HT individuals.

## Methods

### Subjects and ethical approval

One‐hundred and sixteen high tolerant (26 female, 90 male) healthy, normotensive, nonsmoking subjects volunteered to participate in this study (age: 26 ± 8 years; height: 179 ± 11 cm; weight: 77 ± 15 kg; means ± SD), conducted at the US Army Institute of Surgical Research (Fort Sam Houston, TX). This study was conducted under a protocol reviewed and approved by the Institutional Review Board of the Brooke Army Medical Center (Fort Sam Houston, TX) and in accordance with the approved protocol. A complete medical history and physical examination were obtained on each of the potential subjects before being approved for testing. In addition, female subjects underwent a urine pregnancy test within 24 h before experimentation and were excluded if pregnant. Subjects were instructed to maintain their normal sleep pattern and refrain from exercise, alcohol, and stimulants such as caffeine and other nonprescription drugs (i.e., over the counter medications) 24 h before testing to reduce their potential acute effects on cardiovascular responsiveness. During a familiarization session that preceded each experiment, subjects received a verbal briefing and a written description of all procedures and risks associated with the experiments and were made familiar with the laboratory, the protocol, and the procedures. Each subject gave written informed consent to participate in the study.

### Study design

Lower body negative pressure was used as an experimental tool to reduce central blood volume in humans. Application of negative pressure to the lower body (below the iliac crest) results in the distribution of blood away from the upper body (head and heart) to the abdomen and lower extremities, eliciting controlled, experimentally induced hypovolemia. All subjects were instrumented for the noninvasive, continuous measurement of heart rate (HR) via a standard ECG, and beat‐to‐beat arterial pressure via infrared finger plethysmography with a Finometer blood pressure monitor (TNO‐TPD Biomedical Instrumentation, Amsterdam, The Netherlands). An appropriately sized Finometer blood pressure cuff was placed on the middle finger of the left hand, which, in turn, was laid at heart level. Each subject underwent exposure to a LBNP protocol designed to test his or her tolerance to experimentally induced hypotensive hypovolemia.

The LBNP protocol consisted of a 5‐min controlled rest period followed by 5 min of chamber decompression at 15, 30, 45, and 60 mmHg and additional increments of 10 mmHg every 5 min until the onset of hemodynamic decompensation or the completion of 5 min at 100 mmHg. Hemodynamic decompensation was identified by the attending investigator by a precipitous fall in systolic pressure greater than 15 mmHg, progressive diminution of systolic pressure to less than 80 mmHg, bradycardia (fall in heart rate), and/or voluntary subject termination caused by symptoms such as gray out (loss of color vision), tunnel vision, sweating, nausea, or dizziness. All experiments were conducted at room temperature (21.7–24.4°C) and ambient temperature did not change during the experiment (23.05 ± 0.19°C at baseline, 23.09 ± 0.17°C at recovery; *P* = 0.85).

All subjects underwent LBNP to presyncope. SNA was measured directly from the peroneal nerve via microneurography in a subset of males (*N* = 13) and females (*N* = 6). The females were equally distributed in each of the two groups (*N* = 3).

To quantify oscillatory rhythms, nonequidistant beat‐to‐beat data were first interpolated linearly and resampled at a frequency of 5 Hz. Data were then passed through a low‐pass impulse response filter with a cut‐off frequency of 0.5 Hz. Three‐minute datasets were fast Fourier transformed with a Hanning window to obtain power spectra. Spectral power was expressed as the integrated area within the low‐frequency (LF = 0.04–0.15 Hz) and high‐frequency (HF = 0.15–0.4 Hz) ranges for systolic arterial pressure (SAP) and SNA oscillations (HRV‐Task‐Force, 1996); individually identified burst areas were used for SNA oscillation determination. Frequency domain methods assign bands of frequency and then count the number of NN intervals (i.e., normal beats) that match each band. The bands are typically high frequency (HF) from 0.15 to 0.4 Hz, low frequency (LF) from 0.04 to 0.15 Hz, and the very low frequency from 0.0033 to 0.04 Hz. We calculated the unit for SAP–SNA coherence (arbitrary units) by dividing the cross‐spectral densities of the two signals of interest (either SAP or diastolic arterial pressure [DAP] with SNA) by the product of the individual autospectra, and then averaged coherence within the LF range.

### Baroreflex sensitivity calculation

Baroreflex sensitivity (BRS) is a measure of the autonomic nervous system and baroreceptor responsiveness to a stimulus (e.g., change in blood pressure) that results in a modulation in heart rate (HR). In this study, the sequence method described by Gouveia et al. in which spontaneous beat‐to‐beat changes in heart rate are measured as a function of the beat‐to‐beat changes in blood pressure (Gouveia et al. 2008). Briefly, arterial pressure values and R‐R Interval (RRI) were measured beat by beat to give SAP, DAP, and mean arterial pressure (MAP), and the RRI value for each cardiac cycle. These values were used to calculate BRS gains and for power spectral analysis of HR. HR baroreflex gain was determined by least‐square regression analysis of the linear portion of the sigmoid relation between SAP and RRI, when each RRI was plotted as a function of the preceding SAP (one offset). BRS gain was measured from spontaneous sequences of three or more beats when RRI and SAP changed in the same direction using linear regression analysis. Upsequence and downsequence (Down_BRS_) were defined as increasing and decreasing sequences, respectively. Only sequences where successive pressure pulses differed by at least 1 mmHg were selected. Therefore, Down_BRS_ less than 1.0 constitute periods when the minimal standards to calculate BRS with this methodology were not met. Down_BRS_ was autocalculated (WinCPRS) as ∆RRI/∆DAP (ms/mmHg). BRS at the time of decompensation was used to dichotomize subjects into two groups (Group 1 = Down_BRS_ > 1.0, *N* = 49, and Group 2 = Down_BRS_ < 1.0, *N* = 67).

### Calculation of the CRI

As previously described in detail (Convertino et al. 2013, 2016), state‐of‐the‐art feature extraction and machine‐learning techniques were used to collectively process arterial waveforms. Briefly, feature extraction is a form of dimensionality reduction that may be used to facilitate pattern recognition in image and signal processing. Machine learning is concerned with the design and development of models that can be used to automatically extract and integrate task‐relevant information. The combination of these analytical technologies provided a unique computational “tool” to rapidly make sense of very large datasets of waveforms.

For clinical simplicity, the CRI was normalized on a scale of 1–0 (100% to 0%, respectively), where “1” reflected the maximum capacity of the sum total of physiological mechanisms (e.g., baroreflexes, respiration) to compensate for relative deficits in central blood volume, and “0” implied imminent cardiovascular instability and decompensation. Values between “1” and “0” indicated the proportion of compensatory reserve remaining. The model calculated the first CRI value estimate after 30 heartbeats of initialization, and then, in real time, provided a new CRI estimate after every subsequent heartbeat.

It is the physiological basis that drives development of the machine‐learning model. The physiology is based on the premise that the sum total of all compensatory mechanisms involved in the control of cardiac output (ejected wave) and peripheral vascular resistance (reflected wave) are represented by the entirety of features of the total arterial waveform (Convertino et al. 2016). The original model was developed from waveform analog data collected from a finger infrared photoplethysmogram during progressive LBNP experiments conducted on human subjects and validated on a different group of human volunteers (Convertino et al. 2013). The way the algorithm for measuring compensatory reserve works is by evaluating subtle changes in the features of the arterial waveform. The algorithm integrates all of the physiological compensatory mechanisms, and the changes in their status and features in response to compromised circulating blood volume that gives it its unique individual‐specific predictive capability to assess one's capacity to compensate. Each individual's compensatory reserve is correctly estimated because the machine‐learning capability of the algorithm accounts for compromised circulating blood volume. It “learns” and “normalizes” the totality of compensatory mechanisms based on the individual's arterial waveform features.

### Data analysis

Continuous, beat‐to‐beat ECG and Finometer recordings were sampled at 500 Hz and recorded directly to a computer‐based data acquisition software package (WinDAQ; Dataq Instruments, Akron, OH) and then transferred to data analysis software (WinCPRS; Absolute Aliens, Turku, Finland). R waves generated from the ECG signal were detected and marked at their occurrence in time. With the use of the AP waveform as an input, stroke volume (SV) was estimated on a beat‐by‐beat basis using the pulse contour method outlined previously. For the final LBNP level, the last 1 min of data was used for all time domain variables, and the last 3 min of data were used for all frequency domain variables. In the cases where there was less than the required time available during the final level of LBNP, the 1‐min and 3‐min data crossed into the previous LBNP level. Oscillatory patterns of arterial blood pressures were determined with fast Fourier power spectral analysis.

### Statistics

Unpaired *t* tests were used to compare the subject demographic data between the groups. For all data, ANOVA for repeated measures was used for comparison of all physiological variables by Tukey post hoc tests. Unpaired *t* tests were used to compare the two subject populations at 1‐min and 3‐min (frequency analysis) prior to presyncope time points. Unless otherwise stated, all data are presented as (mean ± 95% confidence interval [CI]).

## Results

**Figure 1:**
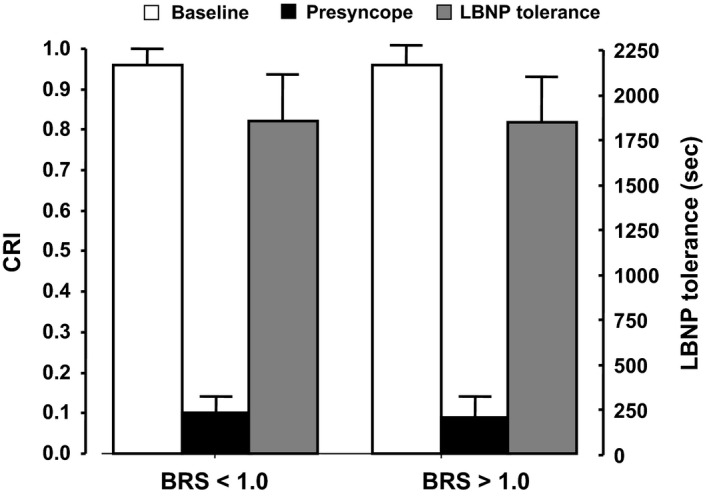
Lower body negative pressure (LBNP) tolerance (gray bars) and compensatory reserve index (CRI) at baseline (white bars) and at the time of presyncope (black bars). Values represent mean (bars) and 95% CI (“T” lines).

The two groups were statistically similar in age, height, weight, and gender distribution. The LBNP protocol was terminated upon completion of −60 mmHg LBNP for 30 subjects, −70 mmHg LBNP for 37 subjects, −80 mmHg LBNP for 31 subjects, −90 mmHg LBNP for 16 subjects, and −100 mmHg for two subjects. As such, 116 subjects were classified as HT. Both groups had similar tolerance times (1849 ± 260 vs. 1839 ± 253 sec) and physiologically similar CRI (0.10 ± 0.03 vs. 0.09 ± 0.01) (Fig. 1).

Hemodynamic and oscillatory responses during LBNP are shown in Table 1. At baseline, MAP, SAP, SV, pulse pressure (PP), cardiac output (CO), total peripheral vascular resistance (PVR), SNA, low‐frequency oscillations in SAP (SAP_LF_), and Down_BRS_ were not different between the two experimental groups (Table 1). Baseline HR was elevated in Down_BRS_ < 1.0 group (66 ± 10 bpm), when compared to the Down_BRS_ > 1.0 group (61 ± 7 bpm).

**Table 1:** Hemodynamic variables at baseline and presyncope

	Baseline		*P* value	Presyncope		*P* value
	BRS < 1.0	BRS ≥ 1.0		BRS < 1.0	BRS ≥ 1.0	
Number of subjects	**49**	**67**		**49**	**67**	
Male/Female	34/15	47/20		34/15	47/20	0.52
HR (bpm)	**66 ± 10**	**61 ± 7**	**<0.01**	**131 ± 20**	**107 ± 19**	**0.007**
MAP (mmHg)	97 ± 8	97 ± 7	0.45	78 ± 11	79 ± 12	0.23
SAP (mmHg)	130 ± 10	131 ± 11	0.29	**93 ± 14**	**98 ± 12**	**0.003**
DAP (mmHg)	76 ± 6	75 ± 6	0.78	69 ± 10	70 ± 10	0.29
SV (mL)	99 ± 23	100 ± 19	0.45	**36 ± 15**	**45 ± 15**	**0.01**
PP (mmHg)	54 ± 9	55 ± 8	0.53	**23 ± 5**	**29 ± 7**	**0.0002**
CO (L/min)	6.4 ± 1.4	6.2 ± 2.2	0.25	4.5 ± 1.2	4.7 ± 1.1	0.41
PVR (mmHg/L/min)	15.8 ± 3.0	16.3 ± 3.8	0.21	**4.5 ± 2.6**	**4.1 ± 1.3**	**0.05**
SNA (B/MIN)	16 ± 4	16 ± 3	0.35	53 ± 7	45 ± 13	0.08
Down_BRS_ (ms/mmHg)	19.5 ± 9.2	19.3 ± 11.3	0.23	**0**	**4.1 ± 4.7**	**N/a**
RRIHF (vagal index)	1439 ± 1449	1685 ± 1542	0.61	**22 ± 58**	**95 ± 130**	**0.002**
SAP LF (mmHg^2^)	7.5 ± 8.5	6.3 ± 8.5	0.23	18.9 ± 14.7	23.7 ± 13.5	0.25
Number of subjects (male/female)	7/3	6/3		7/3	6/3	
SNA (bpm)	16 ± 4	16 ± 3	0.35	53 ± 72	45 ± 13	0.08

Values represent mean and 95% CI (“T” lines). Bold text denotes values that are significantly different.

At presyncope, there were no significant differences between the two groups for MAP (78 ± 12 vs. 78 ± 11 mmHg), CO (4.5 ± 1.2 vs. 4.7 ± 1.1 L/min), and SAP_LF_. Subjects with Down_BRS_ > 1.0 had lower HR (107 ± 19 vs. 131 ± 20 bpm), higher SV (45 ± 15 vs. 36 ± 15 mL), less SNA (45 ± 13 vs. 53 ± 7 bursts/min), and less increase in PVR (4.1 ± 1.3 vs. 4.5 ± 2.6) compared to subjects with Down_BRS_ < 1.0 (Table 1).

## Discussion

There are two primary findings from this study. First, contrary to our hypothesis, higher HR, PVR, SNA, and BRS were not associated with greater tolerance to reduced central blood volume. In a cohort of high‐tolerant subjects, some individuals rely on cardiac filling and vagal withdrawal to defend AP, while others rely on sympathoexcitation to elevate HR and PVR. Second, our data demonstrate that regardless of which physiological mechanisms are used to optimize individual tolerance to progressive reductions in central blood volume, the integrated response of these compensatory changes are accurately reflected in the measurement of compensatory reserve (CRI).

We sought to better understand the relationship between independent and integrated compensatory mechanisms involved in dictating tolerance to reduced central blood volume in a subpopulation of HT individuals. Previous studies have shown that individuals who are high tolerant to reduced central blood volume generally display greater reserves for sympathoexcitation and reductions in BRS (Ley et al. 2009; Convertino et al. 2012), as well as more blood pressure oscillations (Ludbrook et al. 1981; Ryan et al. 2011), and greater vasoconstrictor reserve (Ludwig and Convertino 1994; Convertino et al. 2012), compared with low‐tolerant individuals (Rickards et al. 2009, 2011). Since acute reductions in BRS during lowered central blood volume have been interpreted to reflect baroreflex‐mediated cardiac vagal withdrawal, we tested the hypothesis that subjects with greater reduction in BRS at the time of presyncope would demonstrate greater LBNP tolerance associated with higher HR, PVR, and SNA. While these compensatory physiological mechanisms play an important role in achieving high tolerance to central hypovolemia, we did not observe that each of these parameters is needed in entirety. These results differ from comparisons of compensatory responses to lowered central blood volume to presyncope between LT and HT individuals (Rickards et al. 2009, 2011). The present study is the first to provide comparative data demonstrating the existence of subpopulations with analogous physiological abilities though diverse contributions of compensatory mechanisms to respond to significant reduction in central blood volume.

Based on the results of this study, vagal withdrawal of cardiac baroreflex activation contributes only partially as an important role in achieving high tolerance to central hypovolemia; however, other physiological parameters can compensate for attenuated BRS. In the present study, both of the experimental group baseline values for BRS were similar. However, one group had greater cardiac vagal baroreflex withdrawal (change in BRS) than the other group. Spontaneous baroreflex sequences represent physiological rather than chance interactions between DAP and RRI interval (Bertinieri et al. 1985; Mancia et al. 1986). Specifically, baroreceptor parameters are differentiated in terms of blood pressure elevations (“up” sequences) or blood pressure reductions (“down” sequences). In this study, we used the downward sequence method to assess BRS (Down_BRS_) which has been demonstrated to be more physiologically relevant to reductions in central hypovolemia and stimulated hemorrhage (Cooke and Convertino 2005; Convertino et al. 2012). Additionally, depressed down sequence BRS is associated with several negative clinical health outcomes such as increased incidence of death and malignant ventricular arrhythmias (Pitzalis et al. 1998).

Recently, our laboratory developed an algorithm that accurately tracks the compensatory phase of central volume loss for high‐ and low‐tolerant subjects (Moulton et al. 2013; Convertino et al. 2013, 2016). Our current findings suggest that regardless of which physiological compensatory mechanisms are used to optimize individual tolerance to progressive reductions in central blood volume, these changes are accurately reflected in the CRI. This conclusion is based on evidence that both subpopulations of HT subjects had indistinguishable CRI values that correlated with identical tolerance to reducing in central blood volume loss despite multiple differences in individual compensatory responses. The ability of the CRI algorithm to accurately integrate the various compensatory mechanisms can be attributed to its unique function that analyzes and compares the entirety of features within each arterial waveform. Changes in the waveform features reflect the sum total of all mechanisms that contribute to the cardiac (ejection wave) and vascular (reflection wave) response to varying degrees of central volume loss (Convertino et al. 2013, 2016). Compensatory mechanisms (e.g., tachycardia, vasoconstriction, respiration) are activated by reductions in central blood volume in an effort to maintain perfusion pressure during hemorrhage. CRI represents the integrated capacity of all compensatory mechanisms of any individual to maintain adequate systemic tissue perfusion and thus avoid decompensation (Convertino et al. 2013; Convertino et al. 2000; Moulton et al. 2013; Van Sickle et al. 2013). The present study provides new evidence that CRI has the sensitivity and specificity to integrate the complex and dynamic nature of the physiological mechanisms in an otherwise homogeneous high‐tolerant population.

In summary, the findings of the present study provided unique evidence that higher HR, PVR, SNA, and BRS were not necessarily associated with greater tolerance to  reduced central blood volume. In a cohort of high‐tolerant subjects, some individuals rely on cardiac filling and vagal withdrawal to defend MAP, while others rely on sympathoexcitation to elevate HR and PVR. Importantly, these data demonstrate that regardless of which physiological compensatory strategies are used to optimize individual tolerance to progressive reductions in central blood volume, the integration of these changes are accurately reflected in CRI. We have identified at least two physiological pathways associated with the capacity to compensate for reductions in central blood volume similar to that experienced with hemorrhage leading to cardiovascular decompensation. The primary finding of this study is that CRI accurately reflects the ability to tolerate reductions in central blood volume.

## Disclaimer

The opinions or assertions contained herein are the private views of the authors and are not to be construed as official or as reflecting the views of the Department of the Army or the Department of Defense.

## Conflict of Interest

None declared.
